# General Causes of Disease

**Published:** 1873-06

**Authors:** Wm. Clandenin


### GENERAL CAUSES OF DISEASE.

BY DR. WM. CLANDENIN.

*Read before the American Public Health Association.*

In the enumeration of causes of disease the greatest prominence is given by authors to certain physiological processes—to the establishment and cessation of the functions of certain organs. What a train of childhood's ills is attributed to the evolutions of the primary teeth, and at the age of puberty how many diseases are connected with the catamenial flux, and ater in life to its cessation!

INFANT MORTALITY.

It is humiliating to consider the frightful mortality among children in American cities. One third die in the first year, and one-half before they have attained the fifth anniversary of their birth. This is the more remarkable when taken in connection with the fact that we raise successfully our domestic animals, but fail in rearing our own children.. Where or how shall we seek to discover an explanation of this fact? Are mankind weaker and more fragile than they were once; are they obnoxious to more dangers now than formerly; and have diseases increased in number and severity; or do the requirements of society, the results of our social system, together with the omnipotent behests of custom and fashion, and the various springs put in motion by passion and party spirit, give rise to the constant causes of a more permanent iuterruption of the sense of well being?

Among the various causes assigned for the great mortality of children, "teething" seems to be the most common. Almost every disease of childhood is regarded by professional men the learned and the ignorant, as being the result of pain produced by the eruption of the milk teeth. "The cutting of the milk teeth," Marshall states, "is, doubtless, in many cases, though *not necessarily*, a painful process." If it is not necessarily a painful process, it is necessary to know when or under what circumstances it becomes so. The primary teeth, according to writers on physiology, begin to appear about the seventh month, and are completed at the expiration of the second year.

The statistics of the several health departments of this and of other countries demonstrate that the great mortality among young children occurs during the first six months, dating from birth, and consequently before the teeth begin to appear. The next highest mortality occurs during the last six months of the first year of life. This statement is fully corroborated by the mortality tables of every European country, as well as those of our own. Such tables prove most conclusively that the death rate among children steadily and perceptibly diminishes from and after the fourth and fifth months: that is, after the time at which the teeth generally begin to appear. For example, there were 7,497 children died in New York during the year 1867, whose ages were less than one year; of this number

891 died during the first week, 884 between the first and fourth week, 945 between the fourth and eighth week, 885 during the third month, 1613 between the third and sixth month, and 2,281 during the remaining six months of the year. In Cincinnati, during the last six years, 9,766 were reported to have died under one year of age; of this number 801 died during the first week, 778 between the first and fourth week, 825 between the fourth and eigth week, 703 during the third month, 1,564 between the third and sixth month, and 2,014 during the last half of the year; showing conclusively that the greatest sacrifice of child's life occurs at a period when dentition could not have sufficiently advanced to produce any injurious impression upon the delicate nervous system of the child.

To what causes, then, may we properly and truthfully ascribe this discrepant mortality? And, what is it that so strongly predisposes the child of three months to diseases which one of as many years would successfully resist or escape altogether? Prof. Chaillie, of New Orleans, states that according to the census returns of 1860, in each thousand of the population of the whole United Stqtes there are about 29,07 children under one year of age; and there are about 124,03 in each thousand one year and under five years of age, giving an average for each of the four years of about 31,000 children. In healthy localities the death rate of children under one year of age ought not to exceed one in six; but in some cities of this country it is one to three and a fraction.

Again, we learn from the same authority that the diseases most destructive to life among children under one year of age are the same that, with few exceptions, prove so destructive to life during the succeeding year—viz: Convulsions, congestion, and inflammation of the brain; hydrocephalus, atrophy, and debility; diarrhea, cholera infantum, pneumonia, etc., etc.

### TEETHING.

There are certain physiological changes taking place coincident with teething, but in nowise consequent upon it, which may become pathological. During the early period of childhood the food is taken into the mouth by a sucking process; there is neither mastication nor insalivation, properly so-called; the glands of the stomach and intestines are only sufficiently developed for the digestion of albuminous liquids. Subsequently (and during the period of dentition) the salivary glands enlarge and become active, and hence the cause of the "drooling" so often witnessed, and supposed to be caused by the irritation of dentition ; the glands of the stomach and intestinal tube undergo a simultaneous development, necessary for the digestion of starch and oils. During these changes there is an increased flow of blood to these numerous glands, and under favoring conditions diarrhea and other diseases of the bowels and assimulative organs may ensue.

The popular opinion in reference to dentition as a cause of disease, is often productive of evil consequences. It lessens the appreciation of true causes, and diverts attention from them. If a child is taken sick during the period of teething, the parents, deceived by the popular opinion, attribute the child's illness to that cause, and therefore do not look for, or suspect, the possibility of any other. Thus the real cause of the disease is not, perhaps, discovered at all, or not until it is too late to successfully combat the disease.

Among children the sickness and mortality follows the months, and in cities it is generally increased from fifteen to twenty per cent. by the heat of summer. There is, also, perhaps, a slight increase during the winter months, and yet the mortuary tables, compiled with the greatest attainable accuracy, for many years, prove most positively that on the average of years, and, also, in each single, successive year, the mortality is excessive in cities and large towns as compared with the immediate adjacent country districts under the same climatic conditions. Again, the death rate is never uniform throughout all portions of a city. Those parts which are clean, the houses properly constructed and well ventilated, and where the inhabitants have pure water and good food, show moderate bills of mortality, while, on the other hand, those localities in which the streets are narrow and filthy, and where poor people live in cellars or underground rooms, or are crowded together in tenement buildings, erected, perhaps, upon ground made of the sweepings of streets and market places, and the debris of the city, and where the contents of privies surcharge the porous earth around, the death rate is always much higher, and especially when to these conditions is added high temperature. Wherever these conditions are met with sickness must ensue, and thus not only dentition and other physiological processes incident to growth and development may become abnormal, but if long continued they certainly result in physical deteriorations. Nor can the rich selfishly conclude that they will not be effected by these evils which they allow to scourge the poor.

INFANT'S FOOD.

The hygienic relations of food is a subject that is supposed to be generally understood, and yet nothing in our practical every day life presents so many examples of ignorance, and exhibits such flagrant violations of the laws of health. The history of the Irish famine and pestilence of the years 1846 and 1847 developed the dreadful effects of starvation, and fully demonstrated that the want of food in sufficient quantities to sustain the body is not only a predisposing but actually an exciting cause of disease, and may cause the most terrible epidemics.

The quality and also the adaptation of food to the age and condition of the digestive organs, exercises a powerful influence in the development of disease.

In his treatment of infantile diseases, the medical attendant very seldoms inquires further than to assure himself that his little patient is nourished by its mother, disregarding or forgetful of the fact that the secretion of the mammary glands, both in its quantity and quality, is strongly and directly influenced by the nervous system, and especially by emotional states. Sir A. Cooper affirms that "there is evidence that the mammary secretion may acquire an actually poisonous character, under the influence of violent mental excitement." The same writer states that "Fits of anger produce very irritating milk, followed by griping in the infant, with green stools;" also that "Anxiety of mind diminishes the quantity and alters the quality of the milk." "A fretful temper lessens the quality of milk, makes it thin and serous, and causes it to disturb the child's bowels, producing intestinal fever and much griping." These statements are fully corroborated by other authority.

It is a well known fact that the milk carries with it, to a greater or less degree, the peculiar characteristics of food. Exercise, when excessive, diminishes the quantity of butter, and increases the amount of caseine. What, then, must be the effect upon the child nursed by a mother who is constantly subjected to the harassing and depressing influences of poverty, and its attendant consequences, living, perhaps, in a damp and vitiated atmosphere, upon scanty and unwholesome food?

INHERITED DISEASES.

To these causes must be added the bad consitutions inherited from parents. There are few biologists who dispute the influence of heredity—not only of ancestral peculiarities, but of diseases also. We have sufficient evidence that such a cause does exist, and that it sooner or later in life manifests itself as a depressing influence, and diminishes the power of resistance to disease, and perverts the life processes.

The physician can do little for the inheritors of scrofula, syphilis, and other hereditary diseases, and yet it should be understood that even those thus diseased may outgrow their inherited vices of constitution. Yet, in the language of Maudsley, "Here, as elsewhere in nature, like produces like: and the parent who makes himself a temporary lunatic or idiot by his degrading vice propagates his kind in procreation, and entails on his children the curse of the most hopeless fate."

DOES CIVILIZATION INCREASE THE NUMBER OF DISEASES.

There is a popular opinion, nor confined to the ignorant alone, that as mankind has advanced in a material and intellectual point of view the number of diseases has increased; that many diseases are the necessary accompaniments of mental development, of arts, and industry; and that the higher education of youth produces effeminacy, debility, and disease.

Such a view is incorrect, because every onward step in the path of knowledge and true refinement has had a beneficial influence. The sanitary condition of communities improves, *pari passu*, with the education and intelligence of the people composing it. There are, however, some serious errors connected with and growing out of the manner in which we seek to accomplish the grand purpose of civilization. The principles and plans adopted in most of our schools and colleges is at direct variance with those rules of being and of doing which fulfil all that is needful to bodily health and mental vigor. A uniform standard is adopted by teachers which is usually so high that it is adapted only to the ablest minds, and it has to be attained by the less gifted by undue mental effort. Children are generally classified in schools according to their ages or acquirements at the time of their admission, regardless of their mental capacity or physical state. All the children composing a class are supposed to possess the same vigor and quality of mind, equal powers of application, endurance, and adaptability. Consequently, the powers of the mind of some of the pupils are likely to be overtasked by the constant and severe effort required to keep pace with the curriculum of studies in their classes. From this cause, the brain and nervous system become at first morbidly excitable, but subsequently, if not relieved by rest and relaxation from study, the memory is weakened, and the power of thought and concentration of the mind proportionately diminished. The physical organs promptly sympathizes with, and are speedily brought under the influence of this depressed nervo-mental state, especially when, as is too often the case, large numbers of children are in attendance and kept for so many hours daily in an improperly heated and badly ventilated school building. Both the body and the mind are in this manner very often permanently enfeebled, and nutrition so greatly impaired that growth and development are retarded, perverted, or it may be permanently arrested, and the child is thus doomed to a life of suffering, and perhaps to premature death.

ARE CHANGES IN LIFE DANGEROUS?

The physiological changes which are effected at the period of puberity, and which result in the perfection of the highest physical power of animal life—the power of reproduction, characterized by the awakening of new passions, feelings, and associations—has always been considered as particularly dangerous to life, or as rendering the individual especially susceptible to disease. There is much that, on a hasty survey, seems to countenance such an opinion ; a more careful examination of the question shows, however, the incorrectness of such conclusions. The influences brought to bear, and the new conditions developed, are apparently different in character and effects, but they operate in a similar manner, and produce the same results as those causes to which reference has already been made, in the causation of children's diseases.

CHILDBIRTH..

The proper recurrence of those phenomena associated with ovulation is regarded as evidence of health and vigor of constitution; but if these phenomena be not observed, and the health subsequently becomes impaired, their non-appearance is considered as the cause. The preponderance of females over males in the death list (in the census tables) is assigned by physicians to causes having their origin in derangement in the catamenia ; while on the other hand, those causes which derange or delay the catamenia are likely to be overlooked or wholly disregarded. The whole of the child bearing period is almost universally regarded as peculiarly hazardous to life, and yet we have the authority of Dr. Farr to the point that the " child bearing women of a population are, in the language of the insurance officers, 'select lives.' " And Dr. Brinton, another equally high authority, states that, " Apart from a few exceptions, we are bound to remember that all the perils decreed to the female leave her life, as a whole, rather superior to that of the male of corresponding age ; in other words, that the pain and danger of childbirth do not bring about an excess of mortality at all approaching that which results from the greater exposure, toil, and intemperance of the stronger sex." These statements are fully corroborated by the experience of several life insurance companies of this country. According to the last census, after the thirty-fifth year the death rate of the sexes is in favor of females ; and from fifty-five to sixty nearly one third less women died than men.

### CONSUMPTION AMONG WOMEN.

It seems impossible for physicians to divest themselves of the idea that derangements of menstruation do not cause disease. The fact that consumption is always more fatal among females than males, between the ages of fifteen and thirty years, is again and again brought forward as evidence of the evil effects of deranged catamenia. But those who adduce facts in support of this opinion forget that the requirements of society, so often opposed to reason, and the omnipotent behests of custom and fashion are most fully recognized and courted during this period. The consequences of ovulation are excessively annoying to fashionable women, as it so often interferes with their social pleasures and fashionable amusements. To obviate this annoyance, recourse is often had to ablutions of cold water upon the excited organs ; the consequence is that the flow from the surcharged uterus is immediately arrested by the contraction of the vessels. The same result is oftener unintentionally obtained by the unseasonable change of clothing, or improper and insufficient clothing, thin or light shoes. From these causes, so common and so constant, result many of those diseases which are so prejudicial to female health, and which so often end in consumption.

The local evils which so certainly result from the causes cited are multiform and intricate, as every physician knows, and, grave as they may be, they do not end with the individuals, for it has a moral as well as a physical bearing upon the community. Hematocele, for example, one of the local effects of the sudden arrest of the catamenial flow, may be followed by, or result in, adhesions between the intra-pelvic organs and other organs of the abdomen, or its muscular walls, producing flexion of the uterus, and narrowing or obliteration of its canal, and thus becoming a cause of disease of the uterine functions, preventing conception, or if impregnation should take place, the uterus being bound down by the adhesions referred to, its power of expansion is limited and hence the term of utero-gestation could not be completed, and therefore repeated miscarriages would ensue.

But the most common cause of the excessive death rate among females during the period of greatest activity of the reproductive organs is comprehended in the fact that the greater part of a woman's life is spent in the house or in its immediate surroundings, and hence we look to the condition of the dwelling for the explanation. Thus the mother is subjected to the same conditions—to the same morbific influences—that are mainly instrumental in causing the excessive death rate among young children, vis.: a deteriorated atmosphere, and hemmed in, perhaps, by barriers of filth and want, coupled often with the evils of intemperance. The far-reaching ill effects of these causes fall with immense proportion upon females, and especially upon the poor, the ignorant, and the subordinate. It generally, indeed almost always, falls to the lot of woman to administer to the wants of the sick, and, therefore, she is subjected to the immediate effects of the poisonous exhalations thrown off from the skin and lungs of the diseased; so that when disease is generated in, or imported into, tho dwellings of the poor, it causes neglect of cleanliness, and, therefore, the effects are most likely to manifest themselves in the little children and females, the helplessness of the one and the necessities of the other making it impossible for either to escape.

The same conditions produce the same result in males The report of the Registrar-Generals of England show very conclusively the influence of occupation upon general health and longevity—the death of farmers at the age of 35 to 45 was nine in a thousand, of bakers, fifteen, and of butchers, seventeen. The great mortality among butchers is probably due to the effect of the elements of decaying matter by which they are surrounded in the slaughter house and vicinity. Mr. Lombard exhibits trades in relation to consumption. In 1,000 deaths in each of the different occupations noticed, the following proportions were furnished by this disease:

| | |
|---|---|
| With vegetable and mineral emanations | 176 |
| With various dusts | 145 |

| | |
|---|---|
| With sedentary life | 140 |
| With hot and dry air | 138 |
| With workshop life | 127 |
| With stooping posture | 122 |
| With sudden movement of arms | 116 |
| With muscular exercise and active life | 89 |
| With exercise of the voice | 75 |
| Living in the open air | 73 |

And it may be further stated, that the better the condition of life the less liability to consumption. Marc d'Espine has proved that tuberculosis occasions 68 deaths per thousand among the rich and 233 per thousand among the poor.

